# ^1^H, ^23^Na and ^35^Cl Imaging in Cementitious Materials with NMR

**DOI:** 10.1007/s00723-015-0752-6

**Published:** 2016-02-01

**Authors:** L. Pel, P. A. J. Donkers, K. Kopinga, J. J. Noijen

**Affiliations:** Eindhoven University of Technology, P.O. Box 513, 5600 MB Eindhoven, The Netherlands

## Abstract

A set-up especially designed for semi-simultaneous measurements of ^1^H, ^23^Na and ^35^Cl in ordinary cementitious materials using nuclear magnetic resonance was built. This setup makes use of the main field of a whole body magnetic resonance imaging system (Philips Intera), which has allowed us to combine two measurement setups into one, i.e., a ^23^Na/^35^Cl and a ^1^H insert. This 1.5 T field was chosen as a compromise between the signal-to-noise ratio of the spin-echo signal, which increases at higher frequencies, and the line broadening due to the presence of magnetic impurities of these materials, which leads to a decrease of the resolution at higher magnetic fields. The preliminary experiments show that this setup can be used to the study the interaction of different types of ions with cementitious materials. One-dimensional profiles of the moisture and dissolved ions can be measured with a spatial resolution of about 2 mm for ^1^H, 6 mm for ^23^Na and 9 mm for ^35^Cl.

## Introduction

It is widely acknowledged that chloride-induced corrosion is one of the main degradation mechanisms in civil structures based on reinforced concrete. The corrosion starts as soon as the chloride comes in contact with reinforcement steel bars [1]. The source of chloride can be natural, i.e., sea water, or from de-icing salts. In general the chloride will enter a concrete by advection with moisture or diffusion within the moisture present in concrete. Gaining insight into these transport phenomena can not only improve the assessment of durability aspects of existing structures, but might lead to improved design for new reinforced concrete structures that are to be used in aggressive environments.

At the moment there is lack of experimental data on this topic that can be used to validate or discard the wide range of models available in the literature. Whereas such models might be correct, erroneous input is bound to lead to false predictions (i.e., the garbage in, garbage out principle). Modelling with faulty input parameters might have catastrophic consequences.

There are various methods available to measure ionic chloride content in porous building materials. The most common method is to drill a specimen out of a concrete structure and analyse it chemically in the laboratory (see, e.g., [2]). Traditionally this is done by pulverizing the sample and extracting the soluble chloride content using nitric acid solution. The solution is then analysed for chloride ion concentration using wet chemistry. The most obvious drawback of this method is its destructiveness, but there are more shortcomings, such as its poor spatial resolution and irreproducibility.

Nowadays there is a wide range of non-destructive techniques available [3–5]. While most of these methods are able to detect chloride content non-destructively, none of them is able to measure moisture and ion transport simultaneously. For scientific research a method that can measure combined moisture and ion profiles with high accuracy and high temporal and spatial resolution would be desired as to verify transport models. Moreover, one would like to measure multi ion transport and also to study the interaction of these ions with the materials, where ion exchange can be present.

Despite the low sensitivity of nuclear magnetic resonance (NMR) for ^35^Cl (see Table 1) this method might still be preferred, as it allows to measure different nuclei, ^1^H, ^23^Na and ^35^Cl, simultaneously with a high spatial resolution and give a full insight into dynamic interactions between the ions taking place. In addition, NMR can provide information on the pore-size distribution. Various studies have been reported. Both Yu et al. [6] and Barberon et al. [7] used NMR on solid-solution cement suspensions to look at the binding, where NMR provided both structural and dynamical insight. By Yun et al. [8] a feasibility study was performed for using an NMR sensor to detect ^35^Cl. However, their study showed that this would probably not be feasible for in situ measurements. Cano et al. [9] studied the absorption of 3.4 M NaCl in cement paste using the so-called SPRITE technique.

**Table 1:** Various properties of the nuclei studied by NMR

Nucleus	Spin	Natural abundance (%)	γ/2π (MHz/T)	Relative sensitivity
^1^H	1/2	99.9850	42.5775	1.0000
^23^Na	3/2	100	11.2688	0.0925
^35^Cl	3/2	75.78	4.1765	0.0047

These studies show that it is possible to quantitatively study chloride in cementitious materials using NMR imaging techniques. However, the reported studies on Cl transport are limited often to white cements, whereas ordinary cements always contain magnetic impurities (e.g., Fe), which can influence the relaxation behaviour (see, e.g., [10]). Since the time scale of our experiments covers the region from a few seconds to a few days, our original aim was to build an insert designed for semi-simultaneous measurements of ^1^H, ^23^Na and ^35^Cl in ordinary cementitious materials. As the gyromagnetic ratios and hence the resonance frequencies of the selected nuclei are too far apart to be covered by one insert without seriously compromising the sensitivity, we have chosen to use the main field of a 1.5 T whole body MRI system (Philips Intera). The large space within this system allows us to combine two measurement setups into one, i.e., a ^23^Na/^35^Cl and a ^1^H insert. This 1.5 T field was chosen as a compromise between the signal-to-noise ratio of the spin-echo signal, which increases at higher frequencies, and the line broadening due to the presence of magnetic impurities in these materials, which leads to a decrease of the resolution at higher magnetic fields. We will first discuss the home-built NMR setup, i.e., the multi-nuclei RF electronics and the multi-nuclei insert designed for this purpose. To test the set-up we have looked at the hydration of ordinary cement, i.e., Portland CEM I and blast furnace CEM III, with a 4 m salt solution, where we have focussed on the relation between the ^23^Na and ^35^Cl content/concentration as a function of time, which could not be studied up to now.

## The NMR-Setup

### Multi-Nuclei RF Electronics

**Fig. 1:**
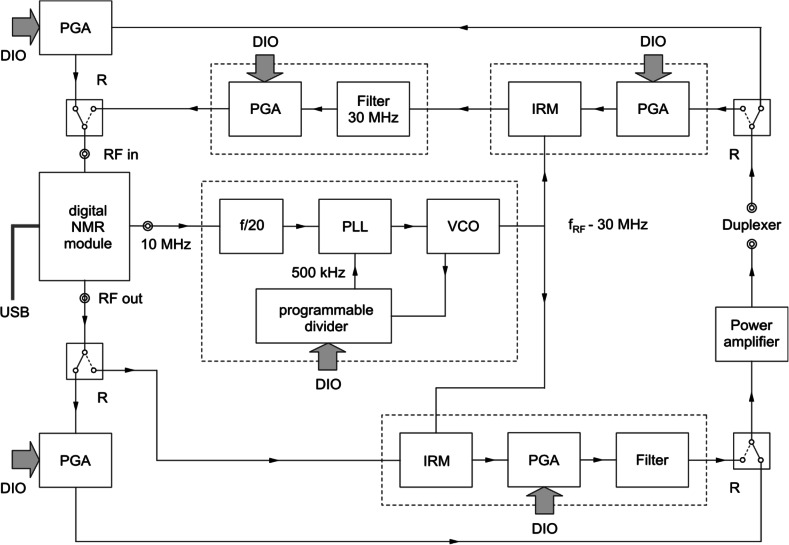
Schematic picture of the multinuclear NMR system

The NMR system used in our experiments is schematically depicted in Fig. 1. The central part of this system is a Radioprocessor^®^ board, manufactured by Spincore Technologies Inc. This digital NMR module can synthesize and sample RF signals up to roughly 35 MHz. It contains facilities for RF pulse modulation and signal averaging, and can be interfaced to a PC via USB. In our applications we perform NMR experiments on different nuclei at various magnetic field strengths, which implies that the frequencies of interest range from about 6 MHz (^35^Cl at 1.5 T) to 200 MHz (^1^H at 4.7 T).

For NMR frequencies below 30 MHz, the RF output signal of the Radioprocessor board is fed into a programmable gain amplifier (PGA), which drives the power amplifier that generates the RF field used to excite the nuclei of interest in the sample. The NMR signal picked up from the sample is amplified by a PGA, before it is supplied to the RF input of the Radioprocessor board. A duplexer isolates the RF signals to and from the sample from each other. The PGA’s are controlled by digital signals via a digital input/output module (DIO) connected to the PC used for experiment control and data acquisition.

For NMR frequencies above 30 MHz, the NMR frequency is shifted to a frequency around 30 MHz, which can be handled by the Radioprocessor board. For this frequency range a variety of RF circuits, such as mixers, is commercially available. The frequency shifting is implemented by mixing the input and output signals of the Radioprocessor board with a signal at a frequency that is a multiple of 2 MHz. The latter signal is created by a voltage controlled oscillator (VCO), which is part of a phase locked loop (PLL). This PLL is synchronized to the 10 MHz reference signal of the Radioprocessor board, which ensures that the phases of all NMR signals are mutually synchronized also.

The RF signal to excite the nuclei is created as follows. The output signal of the Radioprocessor board is mixed with the output signal of the VCO by an image reject mixer (IRM), which significantly attenuates frequencies other than the desired NMR frequency. The output signal of this IRM is amplified by a PGA and filtered to further suppress unwanted frequencies, before it is fed into the RF power amplifier. The NMR signal from the sample is first amplified by a PGA, and subsequently mixed with the output signal of the VCO in an IRM. The output signal of this IRM is filtered by a band pass filter and further amplified by a PGA, before it is fed into the RF input of the Radioprocessor board.

The output frequency of the VCO can be adjusted by changing the divisor of the programmable divider, which is controlled via a DIO connected to the PC. The routing of the signals for NMR frequencies below or above 30 MHz, respectively, is adapted by switching the coaxial relays R, which are also controlled by the DIO module. If needed, some coaxial relays are added to facilitate the use of different RF power amplifiers for different nuclei or different duplexers. The control of all modules in the system depicted in Fig. 1, as well as the data analysis and presentation, is performed by MATLAB^®^ routines.

### NMR Insert

**Fig. 2:**
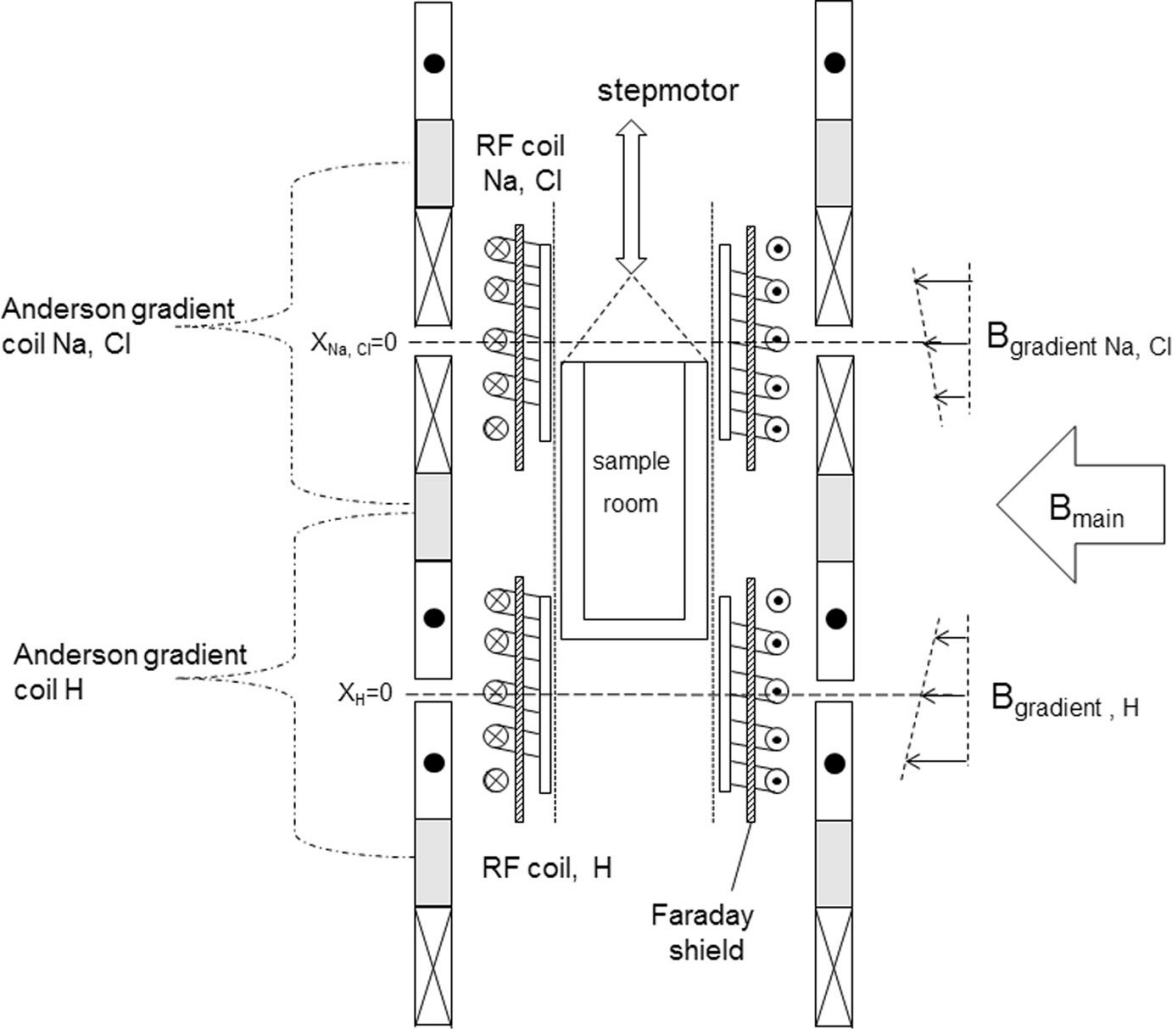
Schematic overview of the home-built insert. A whole-body MRI scanner provides the main magnetic field and the Anderson coils provide the magnetic gradient field. The sample position can be varied and thereby (partially) located in the areas made sensitive to ^1^H or ^23^Na and ^35^Cl NMR. The respective RF circuits contain Faraday shields as to minimize the effect of the changing sample permittivity during the measurements

**Fig. 3:**
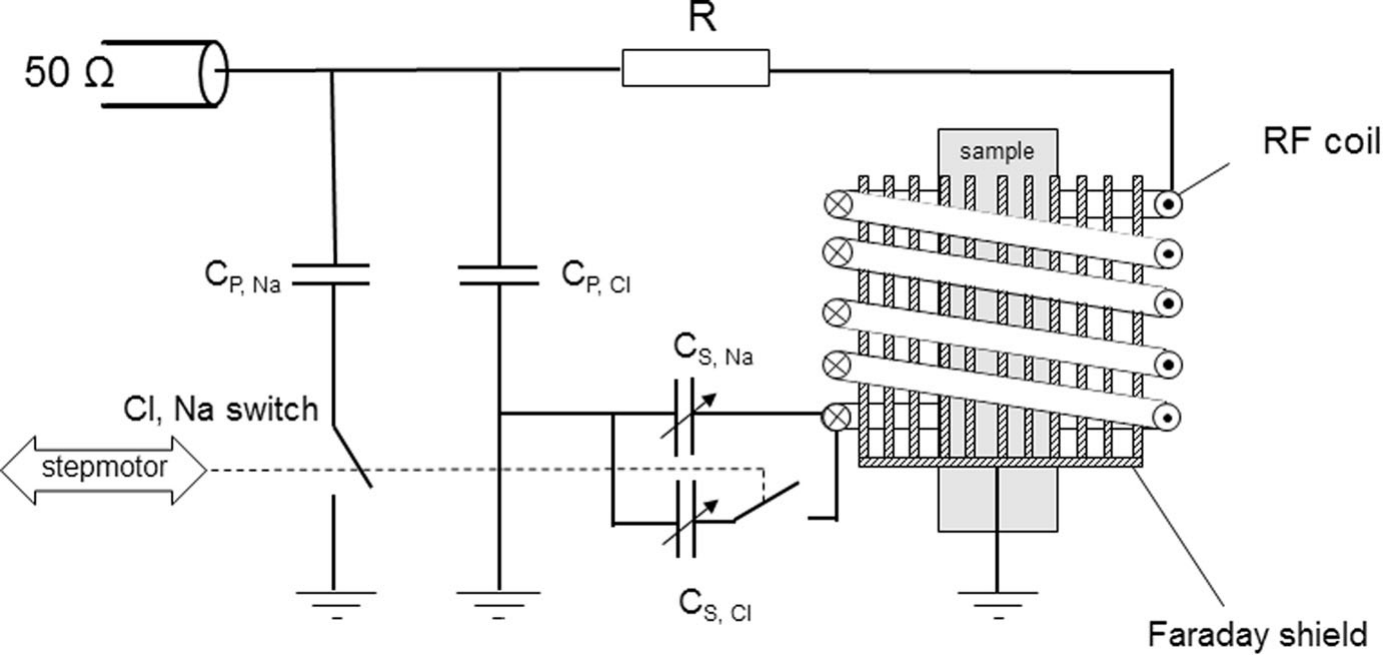
Schematic diagram of the electric resonance circuit of the NMR insert for ^23^Na and ^35^Cl. Using a step motor both the capacitors for the impedance matching and for the resonance frequency can be changed. In this way, the LC circuit can be tuned to the NMR resonance frequency of ^23^Na (33 MHz) or that of ^35^Cl (9 MHz) at 1.5 T. A Faraday shield is incorporated as to minimize the effect of the changing sample permittivity during the measurements

The main 1.5 T magnetic field for the insert is provided by a whole-body medical scanner (Philips Intera). As this medical scanner has a large experimental space, we are able to combine 2 setups operating at different frequencies into one, as schematically depicted in Fig. 2. One part covers the low frequency range for ^23^Na and ^35^Cl measurements, whereas the other covers the high frequencies for ^1^H (and in the future ^7^Li). For an optimum signal-to-noise ratio we chose to use solenoidal RF coils [11] and hence the setup is placed vertically into the main magnetic field of the whole-body scanner. As a result this will limit the maximum length of the sample to be measured. The samples used in our experiments are cylindrical rods with a diameter of 20 mm and a length ranging between 20 and 80 mm. The relatively short RF coils have an inner diameter of 35 mm and a height in the order of 20 mm. The setup contains two RF coils, i.e., the upper one for ^23^Na and ^35^Cl and the lower one for ^1^H, which form part of tuned LC circuits. A specially designed RF circuit was incorporated to be able to switch the frequency of the tuned circuit, of which a schematic diagram is given in Fig. 3. Using two switches which are actuated by a step motor, the capacitors for tuning the resonance frequency and for the impedance matching can be changed. In this way the resonance frequency of the tuned LC circuit can be toggled between 6 MHz for ^35^Cl and 16 MHz for ^23^Na. This step motors is placed well outside the whole-body scanner and is connected to the switches using Bowden cables.

Since our aim is to perform quantitative NMR measurements, special attention was given to the impedance matching of the NMR probe. To reduce the effects of variations of the dielectric permittivity by a changing ^1^H, ^23^Na or ^35^Cl content in a sample, a Faraday shield [12] was added between the LC circuit of the probe head, i.e., the RF coil, and the sample (see Figs. 2, 3). This shield consists of 0.6 mm copper wires running parallel to the axial direction of the coil. The wires are interconnected and grounded at the lower side of the shield, well outside the RF coil. A small slit in this part of the shield prevents the generation of Eddy currents and consequent RF power losses. Care has to be taken in choosing the appropriate wire thickness to prevent acoustic resonance of these wires, which can be picked up as ghost signal. Additionally, the quality factor *Q* of the LC circuit was reduced to about 40 by adding a small series resistor to the RF circuit (see Fig. 3).

To generate a magnetic field gradient 3 pairs of coils are used, which can be combined to Anderson gradient coil sets, either for the upper or lower RF coil. These coils were made from a single plate of 3 mm copper, from which they were cut using a water-jet. The coils have a typical resistance lower than 0.05 Ω. To produce a gradient of 0.15 Tm^−1^ about 50 A are needed and therefore these coils are water cooled. No attempts were made to switch off the field gradients during the individual NMR measurements, i.e., during individual spin-echo sequences. With this gradient of 0.15 Tm^−1^ typically a one-dimensional resolution in the order of 2 mm is obtained for ^1^H, whereas the resolution for ^23^Na and ^35^Cl is respectively in the order of 6 and 9 mm.

The spin-echo experiments are performed at a fixed frequency, corresponding to the centre of one of the RF coils, i.e., corresponding to the centre of the appropriate gradient coil set. The vertical position of the sample can be controlled by a step motor, and hence first a measurement can be performed on the Na/Cl contents, after which the sample is moved over to the position corresponding to the RF coil for H. Again this step motor is placed well outside the whole body scanner and a Bowden cable is used. The spin-echo signal was excited by straightforward (90_*x*_−*τ*−180_*y*_) Hahn pulse sequences. Using the LC circuit described above and a 1.5 KW Tomco^®^ wide-band RF power amplifier (0.5–150 MHz), a 90 degree flip angle of the spins could be achieved with pulses having a duration of 25 μs for ^1^H and 35 μs for both ^23^Na and ^35^Cl.

## Results

To test the performance of the setup we have looked at the interaction of ^23^Na and ^35^Cl with ordinary cement during hydration, i.e., CEM I Portland cement and CEM III Blastfurnace cement. We will first shortly discuss the signal-to-noise ratio of the setup and the measured nuclear relaxation in micro concrete samples. Finally we will discuss the hydration measurements of cement paste with 4 molal (m) NaCl solution.

### Signal-to-Noise Ratio

**Fig. 4:**
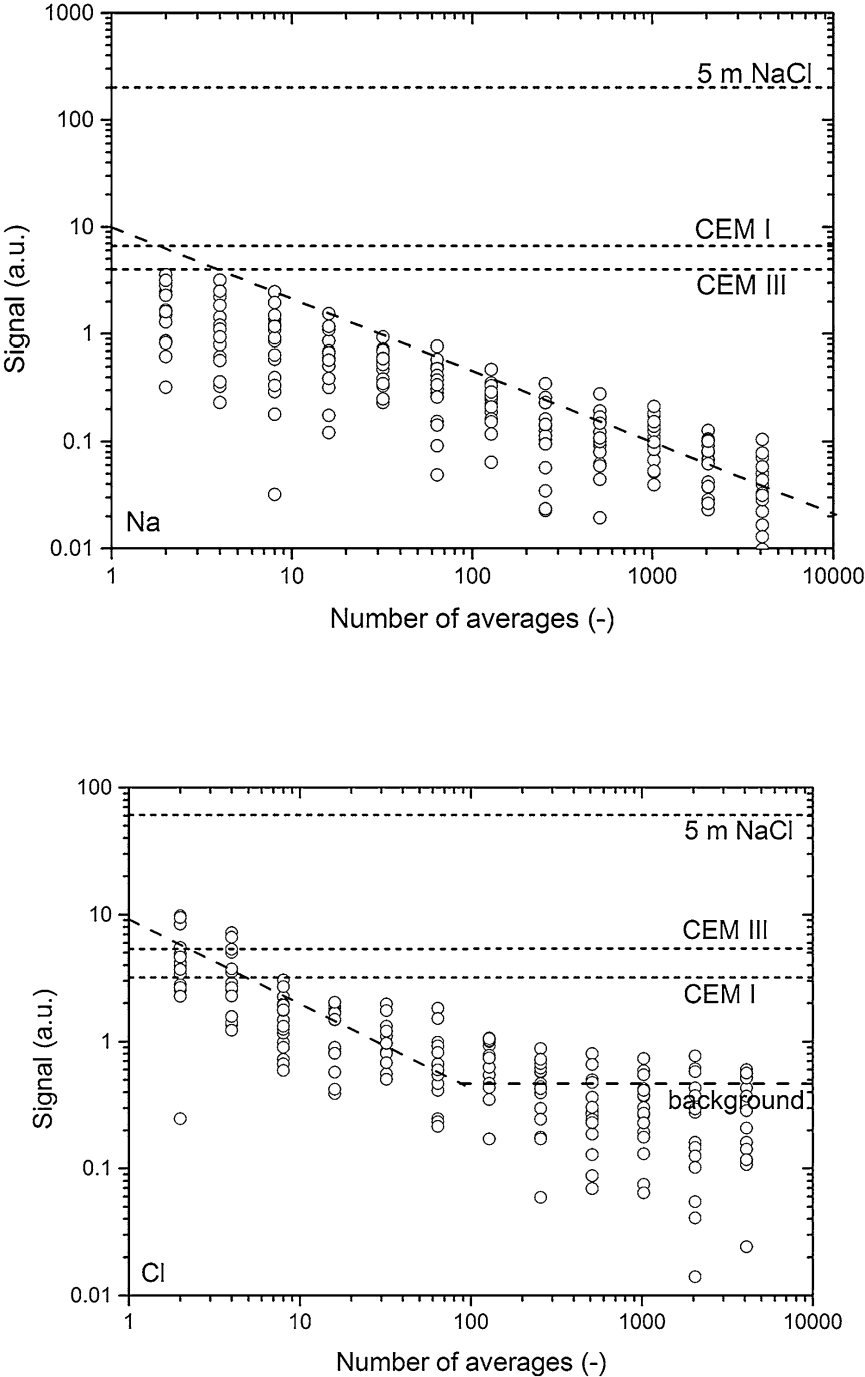
Signal-to-noise ratio as measured for ^23^Na and ^35^Cl. The signal is plotted as function of the number of averages, where each measurement was repeated 8 times. As reference the signals are given for a 20 mm tube filled with 5 m NaCl and 2 cylindrical reference samples of cements paste, saturated with a 4 m NaCl solution, i.e., CEM I and CEM III with *W*/*C* = 0.6

To inspect the performance of the setup, noise levels were measured using a Hahn spin echo sequence, i.e., by measuring the spin echo intensity without placing a sample in the sensitive area of the NMR scanner. This was repeated 8 times for each number of averages. The results for ^23^Na and ^35^Cl are given in Fig. 4. In both cases a clear square root dependence on the number of averages can be seen, as to be expected for random noise. In the case of ^35^Cl a background signal is seen, which we attribute to the various coatings/glues used in the setup. In addition we have plotted the signal for various reference samples, i.e., a 20 mm tube with a 4 m NaCl solution and cylindrical CEM I and III cement pastes samples saturated with 4 m NaCl solution.

### 1D-Resolution

**Fig. 5:**
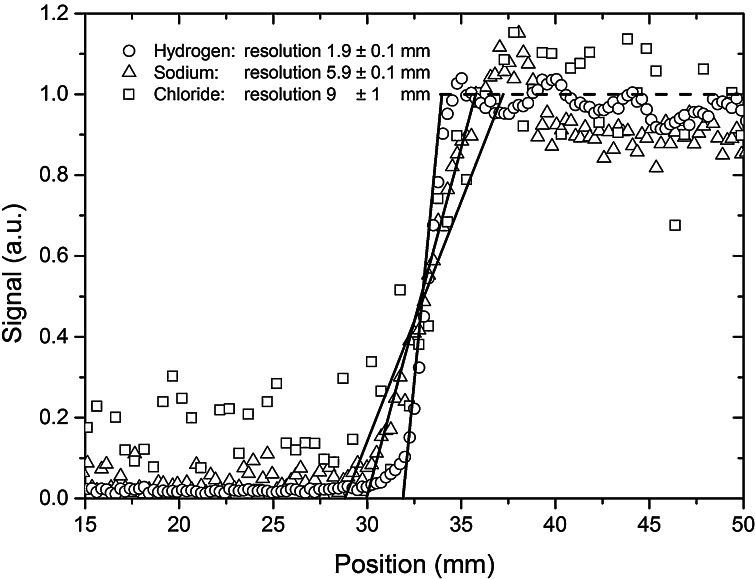
One-dimensional spatial resolution as measured for a cylindrical sample of micro concrete sample (*W*/*C* = 0.6 with max aggregate grains of 6 mm) made of CEM I saturated with a 4 m NaCl solution

To evaluate the performance of the Faraday shield and to determine the 1D resolution for the various nuclei we have measured the profile of cylindrical samples with a flat bottom. Here we have used a micro concrete sample (*W*/*C* = 0.6 with max aggregate grains of 6 mm) made of CEM I This sample was first hydrated for 1 year under water after which is was stored in a 4 m NaCl solution for 1 year. The measured profiles for ^1^H, ^23^Na and ^35^Cl at a constant magnetic gradient of 0.15 T/m are given in Fig. 5. As can be seen in all cases a clear jump is measured indicating there is no strong detuning as intended by the Faraday shield. In addition the resolution is dependent on the gyro-magnetic ratio, i.e., at a constant gradient ^1^H has the highest resolution of 1.9 mm, whereas ^23^Na has almost a 4 times as low resolution of 5.9 mm reflecting the gyromagnetic ratio of the different nuclei given in Table 1. The resolution of ^35^Cl is 9 mm, which was achieved by increasing the window width for recording the echo by a factor of 2 in comparison to ^1^H and ^23^Na.

### Relaxation Behaviour of Ions in Cement

**Fig. 6:**
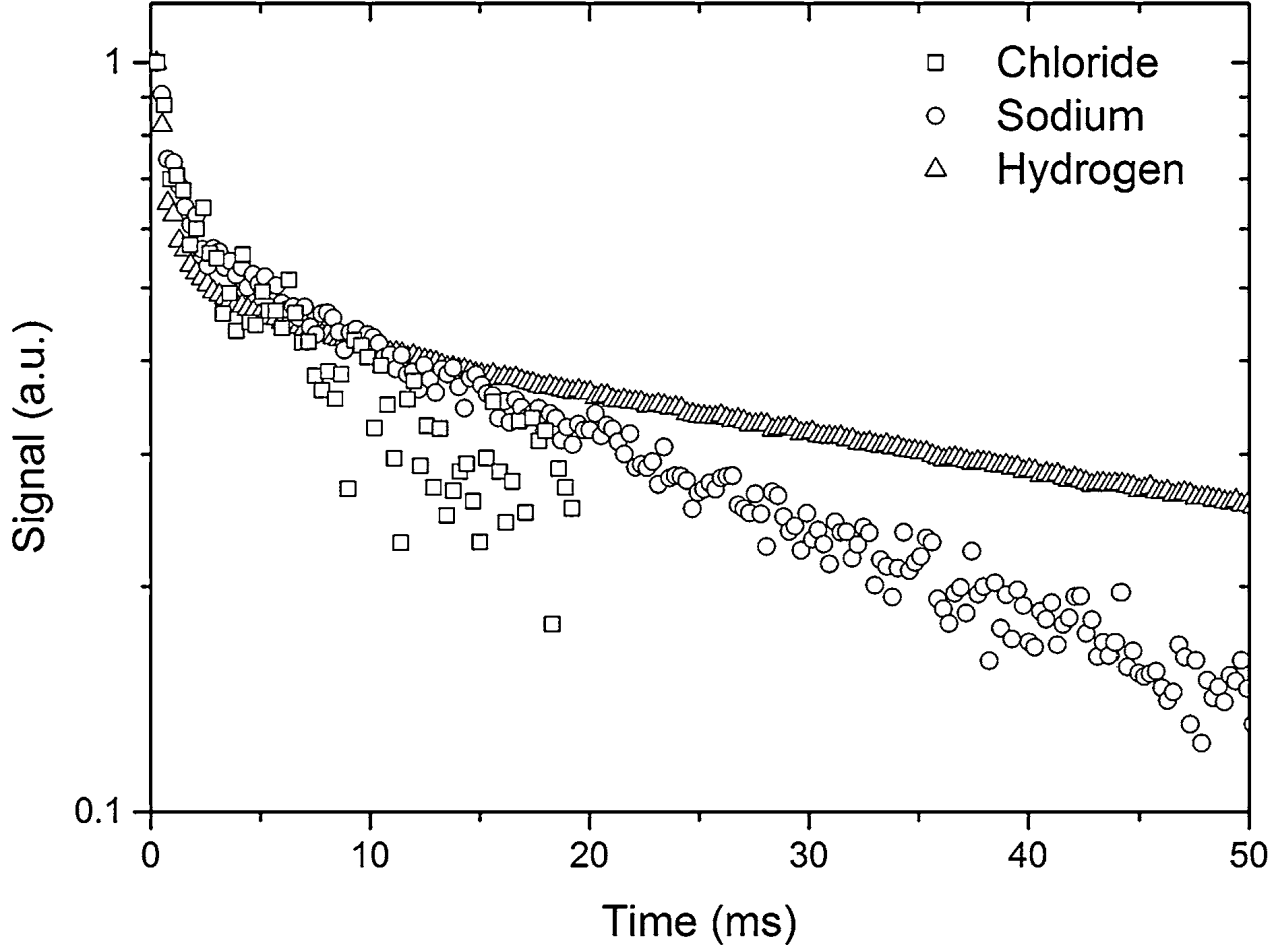
^1^H, ^23^Na and ^35^Cl signals as function of time as measured using a CPMG sequence for a sample of micro concrete sample (*W*/*C* = 0.6 with max aggregate grains of 6 mm) made of CEM I

Next we have applied a CPMG sequence to investigate the transversal (*T*_2_) relaxation of ^1^H, ^23^Na and ^35^Cl in micro concrete sample made of CEM I. After casting, this sample was first stored under water for 1 year after, which it was stored in a 4 m NaCl solution for 1 year as to make certain a stable equilibrium condition was reached. The results are plotted in Fig. 6. For all nuclei a clear double exponential decay is observed. For both ^23^Na and ^35^Cl for longer relaxation time the bulk relaxation will start to dominater for large pores. It has been well established that according to the Brownstein-Tarr model [13] this double exponential decay reflects the gel and capillary pores of CEM I (see, e.g., [14]). Although both ^23^Na and ^35^Cl are quadrupolar nuclei, also here the Brownstein-Tarr model can be applied, as was shown by Rijniers [15]. Since the bulk relaxation time *T*_2_ of both ^23^Na [15] and ^35^Cl [6] is in the order of 30 ms, for cementious materials which have small pores in the order of 10^−9^–10^−6^ m the relaxation behaviour still reflects the pore-size distribution, as the bulk relaxation can be neglected. Indeed, comparing the relaxation curves of ^1^H, ^23^Na and ^35^Cl we see that they all show a similar behaviour for this type of cement. In all cases it can be observed that the ratio between the long and short decay is in the order of 50/50. Using the relaxation time for hydrogen we can determine the pore water distribution. Hence using the relaxation time for ^35^Cl and ^23^Na we could similarly determine the pore ion distribution, i.e., the ion distribution over the pores filled with a NaCl solution and thereby in principle the pore ion concentration.

### Hydration of Cement with a NaCl Solution

**Fig. 7:**
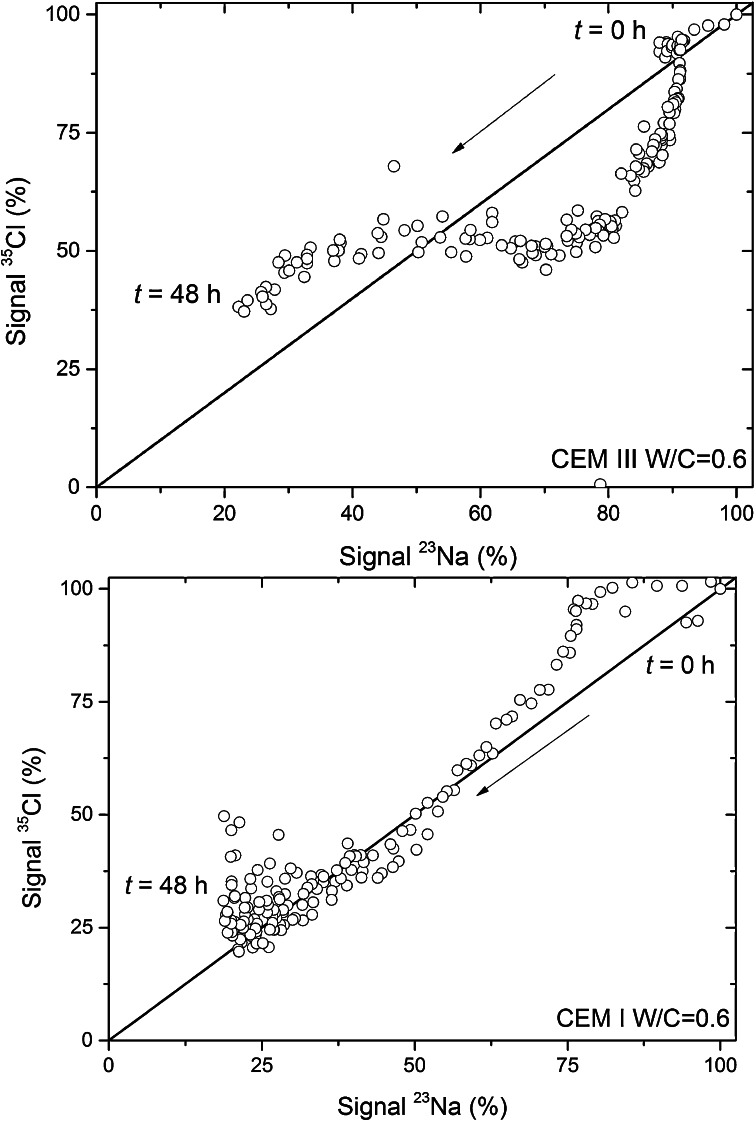
Total measured free ^35^Cl as a function the total measured free ^23^Na in a hydrating cement paste during the first 48 h. Both cements pastes, i.e., Portland CEM I and a blast furnace slag CEM III, were made with a water-to-cement ratio of 0.6 using 4 m NaCl solution

To show the possibilities of the setup we have looked at the interaction of both ^23^Na and ^35^Cl during the hydration of standard cement types, i.e., Portland CEM I and Blastfurnace cement CEM III. With fresh water becoming more scarce, especially in developing countries, there is a tendency to use sea water and hence more information on the effect of salt is needed. During the hydration both types of ions can be chemically bound to cement components, as hydrates are being formed [e.g., Friedel’s salt Ca_2_Al(OH)_6_Cl·2H_2_O]. Especially free chloride ions remaining at the end of the hydration can be dangerous, as they have the capacity to diffuse towards the steel bars of the reinforcement, resulting in corrosion. Of both cement types a paste was made using a 4 m NaCl solution and a water-to-cement ratio (*W*/*C*) of 0.6. These freshly prepared pastes were put into a 20 mm tube, after which immediately the *T*_2_ relaxation was measured for both ^23^Na and ^35^Cl during 48 h. Based on the relaxation analysis using FLI [16] the total amounts of free ^23^Na and ^35^Cl were determined. In Fig. 7 we have plotted the total measured free ^35^Cl as a function the total measured free ^23^Na. For both types of cement initially after the hydration starts, the ^23^Na content immediately decreases, whereas the ^35^Cl content remains almost constant. This shows an interaction with the cement paste where there is an exchange of ^23^Na, and some other ion must come free. For CEM I it is observed that after some time the situation stabilizes and the ^23^Na/^35^Cl ratio becomes almost constant. For CEM III the situation is more complicated. As can be seen the ^23^Na stays almost constant for some time, while the ^35^Cl concentration decreases. Later on this situation reverses and the ^35^Cl concentration is constant while the ^23^Na concentration decreases. This shows that there is a complex exchange of ions during the hydration, which will be studied in more detail in the future.

## Conclusions

Using a specially designed NMR setup, the ^1^H, ^23^Na and ^35^Cl content in cementitious materials can be measured quasi-simultaneously. The sensitivity of the current setup is sufficient to study the intrusion of seawater in cementitious materials. The relaxation of ^1^H, ^23^Na and ^35^Cl can be used to obtain pore-size information, and thereby information on the pore-ion concentration distribution Moreover, the setup has shown that by multi nuclei measurements more insight can be gained on interactions with the cement matrix. It is observed that the Na/Cl ratio changes during the hydration, indication a chemical/physical interaction with the cement matrix.

In the near future we want to improve the signal-to-noise ratio for ^35^Cl by removing any material causing a background signal. We also want to extend the RF-coil for ^1^H as to be able to measure also ^7^Li. This will make the setup also suitable to study alkali silica reactions (ASR), which form also a very important damage mechanism for concrete structures. Moreover, by increasing the sample diameter from 20 to 26 mm, i.e., the maximum diameter allowed within this setup, a 1.7 times increase in the signal-to-noise ratio and thereby a 2.8 times decrease in measurement time can be obtained.
